# Potential of Full-Fat Silkworm-Based Diets for Laying Quails: Performance and Egg Physical Quality

**DOI:** 10.3390/ani13091510

**Published:** 2023-04-29

**Authors:** Yazavinder Singh, Marco Cullere, Davide Bertelli, Severino Segato, Giovanni Franzo, Antonio Frangipane di Regalbono, Paolo Catellani, Cristian Taccioli, Silvia Cappellozza, Antonella Dalle Zotte

**Affiliations:** 1 Department of Animal Medicine, Production and Health, MAPS, University of Padova, Agripolis, Viale dell’Università 16, Legnaro, 35020 Padova, Italy; yazavinder.singh@unipd.it (Y.S.); marco.cullere@unipd.it (M.C.); severino.segato@unipd.it (S.S.); giovanni.franzo@unipd.it (G.F.); antonio.frangipane@unipd.it (A.F.d.R.); paolo.catellani@unipd.it (P.C.); cristian.taccioli@unipd.it (C.T.); 2 Department of Life Sciences, University of Modena and Reggio Emilia, Via Campi 103, 41125 Modena, Italy; davide.bertelli@unimore.it; 3 Sericulture Laboratory, Council for Agricultural Research and Economics, Research Centre for Agriculture and Environment (CREA–AA), Via Eulero 6a, 35143 Padova, Italy; silvia.cappellozza@crea.gov.it

**Keywords:** *Bombyx mori*, *Coturnix japonica*, insect, sustainability, feed, egg production, poultry, egg quality

## Abstract

**Simple Summary:**

Insects can represent a sustainable and alternative feed ingredient to conventional feedstuffs, thanks to a rich nutritional profile, and possibly helping to reduce the feed-food competition. Among various insect species, the mulberry silkworm is of great interest. Silkworm chrysalis is a silk industry by-product, and it is a rich source of high-quality protein and healthy oil. At present, research is required to understand its potential in different food-producing animal species, optimum inclusion levels, and possible side effects. The present research contributes to provide a better understanding of the potential of silkworm chrysalis as a feedstuff for laying quails. The results of the present study demonstrated that the silkworm chrysalis meal could be used in laying quail feed formulation up to 12% inclusion, providing optimal performance and egg physical quality.

**Abstract:**

The present research was conducted to determine the optimal inclusion level of full-fat silkworm chrysalis meal (SWM) into laying quails’ diets, focusing on performance traits and egg physical quality. A total of 240 31-day-old female Japanese quails were randomly assigned to four dietary groups (12 replicates/treatment; 5 quails/replicate); quails were initially fed a standard commercial diet for pullets until 63 days of age. When oviposition started, the experimental groups received the following diets: a conventional corn and soybean-based diet (control diet—C) and three other diets, including 4%, 8%, or 12% of full-fat SWM (SWM4, SWM8, SWM12, respectively). Experimental diets were provided until quails reached 119 days of age. Birds displayed satisfactory productive performance throughout the trial. SWM12 and SWM8 had higher (*p* < 0.001) egg production but also a higher feed conversion ratio compared to C. At the end of the trial, the eggs edible portion increased, and shell weight decreased with increasing the SWM dietary inclusion level (*p* < 0.001). At the same time, SWM12 displayed an increase in albumen pH (*p* < 0.05), even though in the normal range for quail egg. Overall, full-fat SWM (up to 12%) can be considered a promising feed ingredient for laying quails, although higher inclusion levels (>8%) require special attention because SWM also contains anti-nutritional factors.

## 1. Introduction

Lack of animal protein intake has been reported as a critical bottleneck for the population in developing countries, where it remains a concern [1]. Among widely consumed animal products, eggs are a low-cost source of high-quality animal protein, lipids, vitamins, and minerals, thus being suitable for any kind of consumer. Thanks to such positive attributes, egg production is rapidly increasing, and it is expected to reach 95.6 million tons by 2030 [2]. This raises concerns regarding the availability of feed materials in terms of quantity, quality, and continuity. In addition, the increase in poultry production to fulfill animal protein requirements among developed and developing countries is also affecting the cost of raw feedstuffs used for feed preparation, thus impacting livestock production and the cost of their products [3]. The ration for poultry species is typically corn and soy-based and must be supplemented with a sufficient amount of protein and lipids to satisfy the bird growth requirements [4]. In this perspective, insects may be a promising and sustainable feed ingredient to meet the nutritional requirements of poultry species by partially or completely replacing traditional feed ingredients. Among the possible candidates, silkworm (*Bombyx mori* L.) chrysalis is a rich source of nutrients, including high-quality protein and lipids.

Silkworms are reared for silk production, the cocoons formed by silkworms are reeled, and chrysalises are separated. Silkworm chrysalis is a by-product of the silk industry. Occasionally chrysalises are used for food in certain regions of the world; however, chrysalises are often thrown away, leading to a waste of nutrients [5]. From the nutritional point of view, silkworm chrysalis meal (SWM) has a high protein content (50–70%) of high biological value, and lipids are rich in *n*-3 fatty acids, mainly linolenic acid (C18:3 *n*-3), known to be beneficial for human health [6]. Among the bioactive compounds of the silkworm chrysalis, 1-Deoxynojirimycin (1-DNJ) is an α-glucosidase inhibitor, which inhibits glycosidase enzymes through transition-state mimicry [6]. Therefore, enzymes involved in glycogenolysis, glycoprotein processing, and saccharide hydrolysis are inhibited by the 1-DNJ; thus, the possible use of SWM in poultry diets needs to be carefully investigated. Moreover, silkworm chrysalis also contains chitin, a polysaccharide with structural function in many organisms, including arthropods. Previous research findings have shown that chitin can impair nutrient absorption in birds by forming complexes with feed nutrients, mostly proteins and lipids, ultimately reducing their digestibility [7].

So far, available research on SWM as a feed ingredient for poultry is still scarce: most studies considered the inclusion of SWM in broiler diet [6,8,9,10], while the inclusion of SWM in the layer diet has been given less attention. Two studies by Rafiullah et al. [11] and Ullah et al. [12] demonstrated that the gradual replacement (1.4, 2.8, 4.2, and 5.6%) of soybean meal with SWM meal in laying hens’ diet did not affect performance, digestibility, blood profile, egg quality, as well as intestinal health. Diversely, a study by Rahmasari et al. [13] indicated that the inclusion of silkworm meal (from 1.4 to 5.6%) to substitute fish meal (25, 50, and 75%) in diets for laying quails showed a good outcome for the egg production and feed conversion ratio, without negatively impacting the egg quality.

As the optimum inclusion level of SWM in the diet for laying quails has not been established yet, and no upper limits have been identified (yet only limited SWM inclusion levels have been assessed), the objective of the present study is to test different full-fat SWM inclusion levels (4%, 8%, and 12%) into laying quails diet, and evaluate the effects on performance and egg physical quality. Laying quails have been chosen as it is a species of growing economic interest for egg production in different areas of the world as it provides high-quality eggs with a quick return on investment: this is achieved thanks to early sexual maturity, rapid growth, short generation interval, high egg-laying rate, and limited feed and space requirements per bird [14].

## 2. Materials and Methods

### 2.1. Silkworm (Bombyx mori L.) Chrysalis

Oven-dried silkworm cocoons (80 °C for the first 2 h, followed by 6 h at 60 °C) were provided by the Sericulture Laboratory of Padova of the Council for Agriculture Research and Economics, Research Centre for Agriculture and Environment (CREA-AA), Italy. Cocoons were cut open at the Department of Animal Medicine, Production and Health (MAPS), Padova, Italy. Extracted full-fat silkworm chrysalises were ground to a meal at 4000 g for 10 s with a Retsch Grindomix GM 200 mill (Retsch GmbH & Co., Hann, Germany) and used to formulate the experimental diets.

### 2.2. Experimental Design

The experimental trial was conducted on Japanese quails (*Coturnix japonica* L.) at the Azienda Agricola “Il Tramonto” (Casalserugo, Padova, Italy), which has a scientific agreement with the MAPS Department (University of Padova, Padova, Italy). The trial was conducted after being approved by the Ethical Committee of the University of Padova (Protocol n. EC2018/87). For the study, four experimental diets were formulated to meet the minimum energy and nutritional requirement for laying quails [4]: a control diet (C), which was formulated referring to a commercial standard, and three other diets which contained either the 4%, 8%, or 12% of full-fat silkworm chrysalis meal (SWM4, SWM8, and SWM12, respectively). The inclusion levels were chosen considering the results of previously published studies on laying quails [6,13]. Diets were isonitrogenous and isoenergy. The ingredients of the experimental diets are presented in Table 1.

For the experimental trial, a total of 240 31-day-old female quails were individually weighed and wing tagged with individual identification numbers, randomly housed in battery cages (48 cages of 5 quails each; 12 cages per treatment), and fed with a commercial diet for pullets until reaching 63 days of age when egg production reached 70%. On that day, quails were weighed again and assigned to the four treatments such that average initial body weight did not differ between the treatments. The diets and water were provided *ad libitum* throughout the trial. The environmental conditions of the room were monitored and controlled: the average temperature and relative humidity were 21 °C and 76%, respectively, and the adopted photoperiod was 18L: 6D. The birds were fed with the experimental diets for 8 weeks until they reached 119 days of age. 

### 2.3. Productive Performances

Laying quails were individually weighed again at 119 days of age to monitor live weight change during the experimental trial. Weekly feed intake (cage basis) was recorded, and the health status and mortality were also monitored during the trial. During the experimental trial, daily laid eggs/cage were weighed and counted to calculate egg production (Equation (1)), and egg equatorial diameter (mm) and height (mm) were measured using a digital caliper (0–150 mm) to calculate the egg shape index (Equation (2)). Additionally, defective eggs (cracked, without solid shells, or irregular shapes) were counted and used to calculate defected egg percentage (Equation (3)). The feed conversion ratio (FCR) was calculated as kg of feed consumed/kg of egg produced.

(1) $$Egg\;production,\;\%=\left(\frac{number\;of\;laid\;eggs}{number\;of\;quails}\right)\times 100$$

(2) $$Egg\;shape\;index,\;\%=\left(\frac{equitorial\;diameter\;\left(\mathrm{mm}\right)}{height\;\left(\mathrm{mm}\right)}\right)\times 100$$

(3) $$Defected\;egg,\;\%=\left(\frac{defected\;egg\;laid}{Total\;egg\;laid}\right)\times 100$$

### 2.4. Egg Physical Analyses

During week 1, a total of 84 eggs were sampled (21 eggs/treatment), and during week 8, a total of 336 eggs were sampled (84 eggs/treatment) and transported to the LabCNX laboratory of the MAPS Department (University of Padova) and assigned to the following determination: egg weight to calculate surface area (Equation (4)).

(4) $$Surface\;area,{\;\mathrm{cm}}^{2}=3.9782\times {\left(egg\;weight\right)}^{0.7056}$$

After the above-mentioned physical measurements, the eggs were broken for shell and interior egg quality measurements. Within 30 s, albumen height was measured with a Haugh digital micrometer (Baxlo, Barcelona, Spain), and the arithmetic mean of two measurements/egg was performed to calculate the Haugh unit (HU) (Equation (5)). Thereafter, yolk color was evaluated with the 15-point Roche Yolk Color Fan (DSM, Wurmisweg 576, CH-4303 Kaiseraugst, Switzerland). The eggshell was dried with a paper towel and weighed to compute shell percentage (Equation (6)), then eggshell thickness (mm) was measured at the equatorial level with the above-mentioned digital caliper. The weight of the egg and that of the edible portion were used to obtain the edible portion percentage (Equation (7)).

Similarly, a total of 336 eggs were sampled (84 eggs/treatment) during the 8th week of the trial and assigned to physical analyses following the same method described for week 1. In addition, yolk and albumen weights were determined to compute yolk percentage (Equation (8)) and albumen percentage (Equation (9)), as well as yolk to albumen ratio. The pH of albumen was determined with a portable pH meter (FG2-Five GoTM; Mettler Toledo, Greifensee, Switzerland) calibrated at pH 4, 7, and 10.

(5) $$Haugh\;unit:(100\times \log\left(albumen\;height\;\left(\mathrm{mm}\right)-1.7\times egg\;weight\;{\left(g\right)}^{0.37}+7.57\right))$$

(6) $$Shell\;percentage,\;\%=\left(\frac{shell\;weight\;\left(g\right)}{egg\;weight\left(g\right)}\right)\times 100$$

(7) $$Edible\;portion,\;\%=\left(\frac{egg\;weight\;\left(g\right)-shell\;weight\;\left(g\right)}{egg\;weight\left(g\right)}\right)\times 100$$

(8) $$Yolk\;percentage,\;\%=\left(\frac{yolk\;weight\;\left(g\right)}{egg\;weight\;\left(g\right)}\right)\times 100$$

(9) $$Albumen\;percentage,\;\%:\left(\frac{albumen\;weight\;\left(g\right)}{egg\;weight\left(g\right)}\right)\times 100$$

### 2.5. Chemical Analyses

The chemical composition and energy content of the full-fat SWM and the experimental diets are provided in Table 2. Analyses of the SWM and the experimental diets were performed in duplicate following the Association of Official Analytical Chemistry [15] methods to determine dry matter (DM; method no. 934.01), crude protein (CP; method no. 2001.11), and ash (method no. 942.05). Chitin content was determined according to the method described by Zhang and Zhu [16], with the modifications provided by Woods et al. [17]. The ether extract was determined after acid hydrolysis [18]. Starch content was analyzed only in the experimental diets (method no. 996.11). The 1-Deoxynojirimycin (1-DNJ) content was determined by a method developed by Wang et al. [19] and Vichasilp et al. [7], with the modifications provided by Dalle Zotte et al. [6]. An adiabatic bomb calorimeter was used to measure gross energy [20]. For the experimental diets, the calcium and phosphorus analyses were performed by ICP-OES (Spectro Ciros Vision EOP) after microwave digestion (AOAC [15]: method no. 968.08 and 995.11, respectively). The amino acid, mineral, and fatty acid profiles of the SWM can be found in the study by Khan et al. [10] and Dalle Zotte et al. [6].

### 2.6. Statistical Analysis

Data obtained in the present study were subjected to two different one-way analyses of variance (ANOVA) with the experimental diet (C vs. SWM4 vs. SWM8 vs. SWM12) as a fixed effect, following the general linear model (Proc GLM) procedures of the SAS (SAS^®^ OnDemand for Academics—3.81 Enterprise Edition, SAS Institute Inc., Cary, NC, USA). For performance data, the experimental unit was the cage, while for egg physical traits, the experimental unit was the single egg. Least square means were obtained using a Bonferroni adjustment: *p*  <  0.05, *p*  <  0.01, and *p*  <  0.001 were assigned as significance levels.

## 3. Results

### 3.1. Productive Performances

The results of weekly egg production are presented in Table 3. The dietary inclusion of full-fat SWM into the laying quails’ diets improved overall egg production (weeks 1–8), with a linear increase following the SWM inclusion level (4%, 8%, and 12%). Specifically, quails fed with SWM12 (87%) displayed a higher egg production compared to C (84.1%), whereas SWM8 (86.8%) and SWM4 (86.3%) displayed intermediate values (*p* < 0.01). Additionally, egg production for SWM8 and C differed at a lower significant level (*p* < 0.05). Considering the single weeks, the only significant results were highlighted at weeks 2 (*p* < 0.01) and 7 (*p* < 0.05). In the first case, SWM8 and SWM12 showed a higher egg production than the C group, with SWM4 being intermediate. Differently, at week 7, differences reflected those highlighted by the global scenario (weeks 1–8). 

The effect of the dietary inclusion with 4%, 8%, and 12% full-fat SWM on the weekly egg weight is shown in Table 4. The overall (weeks 1–8) egg weight was on the significant threshold but not affected by the dietary treatments (*p* = 0.0587). However, considering single weeks, significant differences were observed at weeks 1 (*p* < 0.01), 2 (*p* < 0.001), 3 (*p* < 0.001), 4 (*p* < 0.001), 6 (*p* < 0.01), and 7 (*p* < 0.001). In these weeks, the groups SWM8 and SWM4 provided the best outcomes having an average egg weight of 13.7 g compared to the SWM4 (13.6 g), C (13.5 g), and SWM12 (13.4 g) groups. 

Results presented in Table 5 show that, overall, the dietary inclusion of full-fat SWM worsened the FCR when the dietary inclusion levels were 8% and 12%. Specifically, the global (weeks 1–8) FCR was significantly higher for SWM12 (3.24) and SWM8 (3.13) compared to the C (2.74), whereas SWM4 (3.00) displayed an intermediate value (*p* < 0.001). A similar trend was also observed in weeks 3, 4, 5, 6, 7, and 8, where SWM12 and SWM8 displayed higher, thus worst, FCR compared to the C group. Differently, SWM4 remained intermediate. 

### 3.2. Egg Physical Traits

The effect of the dietary inclusion with 4%, 8%, and 12% full-fat SWM on the weekly egg shape index is presented in Table 6. Independent of the inclusion level, laying quails fed a diet containing full-fat SWM produced more elongated and ovoidal eggs compared to the C group (*p* < 0.001). In fact, the global egg shape index was significantly lower in the SWM12 (75.8%) group compared to SWM8 (76.3%) and SWM4 (76.3%) groups, which were similar among each other, whereas the highest egg shape index was recorded for the C (77.2%) group (*p* < 0.001). In the case of the weekly egg shape index, a similar trend was recorded throughout the experimental trial (*p* < 0.001).

The physical traits of quail eggs collected at the beginning of the experiment (63 days of age) were similar (*p* > 0.05) in all treatment groups (Table 7). The sole exception was the yolk color FAN, which showed a higher value in the C group compared to SWM4 and SWM12 ones (*p* < 0.01), with SWM8 being intermediate.

However, at the end of the experiment and thus when eggs were collected and analyzed during the 8th week of the trial, the scenario changed as different traits were influenced by the dietary treatments (Table 8). The egg edible portion was significantly higher for SWM12 (88.7%) and SWM8 (88.4%) compared to the C (87.6%) group, while SWM4 (88.3%) displayed an intermediate value (*p* < 0.001). Oppositely, lower eggshell weight was recorded for SWM12 (1.52 g) group compared to the C (1.68 g), whereas SWM4 (1.62 g) and SWM8 (1.61 g) displayed intermediate values (*p* < 0.001). However, SWM12 and SWM4 displayed significant differences at lower levels (*p* < 0.05). Such results coherently affected the shell percentage in a similar manner: it was higher in the C group (12.4%) compared to SWM12 (11.3%) and SWM8 (11.6%), whereas SWM4 (11.7%) showed an intermediate value (*p* < 0.001). Differently, the egg surface area, yolk-to-albumen ratio, and shell thickness remained similar in all groups (*p* > 0.05).

Overall, results on the egg albumen traits such as albumen weight (*p* > 0.05), albumen percentage (*p* > 0.05), and HU (*p* > 0.05) were not affected by the dietary inclusion of full-fat SWM into the laying quails’ diet, with the exception of the albumen pH. The albumen of SWM12 eggs was higher (9.13) than those of SWM8 (9.07) and C (9.07) groups, with an intermediate value displayed in the case of SWM4 (9.10) eggs (*p* < 0.05).

Similar to the observed results on albumen traits, most yolk traits (the yolk weight and yolk percentage) were not affected by the SWM dietary inclusion (*p* > 0.05). The only exception was the yolk color FAN, which was significantly higher in the SWM12 (4.99) and C (5.01) eggs than in the SWM8 (4.62) ones, whereas SWM4 (4.83) was similar to all groups. 

## 4. Discussion

In the present study, quail egg production was observed to improve with the increase of the full-fat SWM dietary incorporation. This result could be attributable to the high quality of SWM protein, characterized by a high content of lysine and methionine (essential amino acids), compared to the other conventional feed ingredients, such as soybean meal [23]. Similar findings were also reported by Rahmasari et al. [13], where the highest inclusion level (2–6%) of SWM into the diet for laying quails increased egg production. Conversely, in another research on laying hens, increasing the inclusion level up to 6% did not affect egg production [11,12]. Together with the protein quality, the dietary lipids profile could have played a role in this result since the equilibrium between saturated fatty acids (SFA) and unsaturated fatty acids (UFA) was reported to influence ovulation. In fact, UFA such as eicosapentaenoic acid (EPA, C20:5, *n*-3) and arachidonic acid (AA, C20:4 *n*-6) are precursors of prostaglandins, leukotriene, thromboxane, and lipoxins, and the dietary intake of *n*-3 and *n*-6 fatty acids can influence prostaglandins synthesis and metabolism [24]. The changed pattern of prostaglandins synthesis may affect the reproductive functions (e.g., ovulation) by affecting the hormonal secretions: follicle stimulation hormone (FSH) and luteinizing hormone (LH) [25]. Since full-fat SWM is rich in *n*-3 FA, it was expected to change the FA profile of the diets (data not shown) and thus possibly affect hormonal secretions. 

Independently of the dietary treatments, the eggs laid by quails in the present study were averagely heavier (13.7 g) than values commonly found in the literature for this species [13]. Egg weight is associated with the quail genotype, and eggs from meat-type quails (13.1 g) are generally reported to be heavier than those produced by laying quails (11.5 g) [26]. There are several other factors that can affect the egg weight, i.e., weight and age of the bird, dietary fat, and protein contents. During the trial (weeks 1, 2, 3, 4, 6, and 7), continuous variations in the egg weight were observed in the different dietary treatments. However, no clear patterns were identified since the different groups alternatively displayed higher or lower egg weight depending on the considered week. For this reason, the overall egg weight (weeks 1–8) was similar in all groups of quails (Table 4). The latter result was expected since all quails had the same age, were purchased from the same incubator, and diets were isonitrogenous and isoenergy. 

Feeding increasing levels of full-fat SWM to laying quails determined a progressive increase in the feed conversion ratio (FCR), which was higher in SWM8 and SWM12 quails compared to those of the C group (Table 5). On the one hand, an increase in egg production of groups fed higher levels of SWM could have determined higher nutrient requirements and, thus, higher feed intake [13]. However, it is also known that SWM contains anti-nutritional compounds such as chitin (polysaccharide) and 1-Deoxynojirimycin (1-DNJ), which is an α-glucosidase inhibitor: both compounds partially inhibit nutrient absorption, either by forming complexes with nutrients available in the gastrointestinal tract of the bird, or by competitively inhibiting specific enzymes involved in glycogenolysis, glycoprotein, and saccharides hydrolysis [6,7]. It was probably mainly for this reason that quails fed with dietary inclusion of SWM above the 4% threshold exhibited a worsening in the FCR, although the same groups had also shown higher egg production (Table 3). Another possibility, yet to be carefully investigated, is a simple feed-choice preference for SWM diets based on sensory cues. Even if no studies on laying quails fed with insects have been focusing on this aspect up to now, research testing the dietary inclusion of a 12.5% full-fat or defatted SWM into broiler quails’ diets, observed a marked reduction in feed choice, which was attributed to the excessive dietary amount of SWM for this bird species at this growth stage [6]. Research on Japanese quail fed with different insect species found heterogeneous outcomes: when *Hermetia illucens* larvae were incorporated in the diets for broiler quails (15%), a comparable feed-choice result to the control diet was observed [27], whereas Woods et al. [17] found a lower feed preference in quails fed with a 10% inclusion *Hermetia illucens* larvae, with the sole difference in the two studies being the inclusion levels and the different substrates used to rear larvae. Differently, Hatab et al. [28] highlighted that a 5 or 10% *Spodoptera littoralis* dietary incorporation reduced feed intake and improved FCR in broiler quails, but no direct feed-choice evaluation was carried out. Previous studies on laying hens highlighted that FCR remained comparable to the group-fed control diet [11,12], but the maximum inclusion level (6%) was lower than the two groups of the present study exhibiting a FCR increase. A result opposite to that observed in the present study was highlighted by Rahmasari et al. [13], where laying quails fed with growing inclusion levels of SWM (2.08–6.25%) observed a reduction in FCR compared to the group fed with a control diet. In this case, the result could be attributable to the different dietary fat amounts in the control diet (higher: 6.71%) vs. those containing SWM (lower: 3.67–4.81%).

Although the inclusion of full-fat SWM modified the egg shape index compared to the C diet, results were in line with values reported for laying quail [14] and close to the normal range, i.e., 72–76%, indicated for hens eggs [29]. In laying quails, the egg shape index can be influenced by a variety of factors, including genetics, age, nutrition, and environmental conditions [30,31]. Even though the above factors were similar among experimental groups, a progressive reduction for this index with increasing SWM incorporation levels was observed, which remains to be understood and justified, additionally, because this is the first time that such an effect was observed and no other studies considering SWM and laying quails have considered this trait up to now.

At the beginning of the experiment (63 days of age), quail eggs exhibited similar physical traits, which was expected since groups had just been created, and, up to that moment, all birds had consumed the same commercial diet for pullets. Therefore, even the different yolk color observed in C, SWM4, and SWM12 groups was not attributable to the diet. Observing the physical egg quality at the end of the experiment (119 days of age), the higher edible portion of SWM8 and SWM12 eggs compared to the C ones was linked to the lower weight, and thus incidence, of the eggshell. 

The main constituent of the eggshell is calcium (93–94% CaCO_3_) and, to a lesser extent, zinc, phosphorous, iron, manganese, etc. It can be hypothesized that the four diets, despite having a similar Ca and P or Ca/P ratio, may have a different bioavailability of minerals due to different content of feed ingredients such as corn, soybean, wheat, silkworm, etc. In this regard, plant-based feed ingredients (oilseeds, legumes, etc.) contain phytic acid (negatively charged molecule), able to form insoluble complexes with positively charged molecules, such as mineral cations (calcium, iron, manganese, etc.), thus decreasing their bioavailability [32]. Furthermore, as discussed earlier, SWM contains chitin, and its deacetylation during digestion forms chitosan, a compound responsible for reducing nutrient absorption in the gastrointestinal tract of birds, including calcium [33]. These two factors could thus be responsible for the reduced eggshell weight and, thus, the incidence in SWM-fed quails of the present study. 

The pH of a fresh quail egg is generally higher (8.00–9.2) [14] than that of a fresh hen egg (7.7–8.5) [34]. The pH value of albumen indicates the freshness of the egg. As the eggs were analyzed on the same day when laid, different values were not expected in the experimental groups. However, SWM12 eggs displayed a higher pH value, even though values were in the normal range for this species. Another indicator of egg freshness is the Haugh unit (HU), which remained comparable among the experimental groups, therefore not raising particular issues regarding the finding about pH and demonstrating that the inclusion of full-fat SWM (up to 12%) does not affect the quality of the eggs. The HU values in this study are comprised of between 94.0 and 94.7, in accordance with what was observed by Rahmasari et al. [13]. Furthermore, the eggs in the present study could be classified in the best AA grade category according to the classification made for chicken eggs by Caner and Yüceer [35]: HU: >72 is categorized as AA grade, between 72 and 60 (A grade), between 59 and 31 (B grade), and <30 (C grade).

The factors that typically influence the yolk color are the pigments (xanthophylls, carotenoids, etc.) naturally contained in the ingredients of the diet in the absence of synthetic xanthophylls. The presence of synthetic pigments is the reason why a more intense yolk color was observed at the beginning of the study (commercial diet with added pigments) compared to that measured at the end of the experiment (experimental diets without added pigments). Naturally, corn is the main source of pigments that are then incorporated into the yolk, thanks to the presence of zeaxanthin and lutein. The highest SWM inclusion level (SWM12) displayed similar yolk color compared to the eggs from the C group; however, the two diets differed in corn content, higher for C than SWM12 group. However, also SWM contains pigments such as beta-carotene and lutein [36], which may have counterbalanced the lower proportion of corn in the SWM12 diet. By looking at the literature results regarding yolk color, a great variability in absolute values can be observed: eggs obtained from quails-fed black soldier fly larvae meal (up to 15%) [14] showed similar values to those reported in the present study. However, Rahmasari et al. [13] found a more intense yolk color (Color FAN 6.16) when full-fat SWM (up to 12% vs. up to 6%) was incorporated into laying quails diets as a substitute for fishmeal, thus highlighting once more the key role of feed ingredients (and thus their pigments content) into yolk color formation.

## 5. Conclusions

The present study has displayed promising results regarding the possible use of full-fat silkworm (*Bombyx mori*) chrysalis meal as an alternative ingredient to the conventional feed sources used in poultry farming, which could also represent an innovative differentiation element, yet sustainable, for the feed industry. Overall, full-fat SWM proved to be an excellent feed ingredient for laying quail at the tested inclusion levels (i.e., 4%, 8%, and 12%), able to improve egg production without determining relevant modifications to the physical egg quality traits. The only aspect that deserves particular attention is the negative effect exerted by the 8% and 12% full-fat SWM inclusion levels on the FCR, attributable to the anti-nutritional factors (chitin and 1-deoxinojirimycin) typically present in mulberry silkworms. Further results considering the sensory traits and nutritional quality of quail eggs will provide a broader indication regarding the commercial potential of this innovative feed ingredient, given that SWM oil is rich in omega-3 fatty acids, whose relevance for human health is well known. 

## Figures and Tables

**Table 1:** Ingredients of the experimental diets (g/kg, as is) ^1^.

Ingredients	Experimental Diets			
	C	SWM4	SWM8	SWM12
Corn	474	481	449	369
Soybean meal	136	214	229	179
Soybean oil	267	101	10.0	0.00
Wheat flour	0.00	32.4	45.1	45.2
Wheat bran	47.3	57.8	112	211
Full-fat silkworm chrysalis meal	0.00	40.0	80.0	120
Calcium carbonate	61.4	61.8	60.9	59.3
Dicalcium phosphate	4.80	3.30	4.00	7.10
Methionine DL	1.60	0.60	0.00	0.00
Lysine	0.00	0.00	0.00	0.00
Vitamin-Mineral premix ^2^	5.00	5.00	5.00	5.00
Salt (NaCl)	3.50	3.50	3.50	3.50
Total	1000	1000	1000	1000

C: Control diet; SWM4, SWM8, and SWM12 are diets corresponding to 4%, 8%, and 12% silkworm (*Bombyx mori*) chrysalis meal inclusion levels, respectively; ^1^ The nutritional value of the diets was calculated according to the Institut National del la Recherche Agronomique (INRA) procedures by using the analytical composition of the raw materials which were provided by the feeding company; ^2^ Vitamin and mineral premix provided the following per kg of diet: Vitamin A, 11,500 IU; cholecalciferol, 2100 IU; vitamin E (from dl-tocopherylacetate), 22 IU; vitamin B12, 0.60 mg; riboflavin, 4.4 mg; nicotinamide, 40 mg; calcium pantothenate, 35 mg; menadione (from menadione dimethyl-pyrimidinol), 1.50 mg; folic acid, 0.80 mg; thiamine, 3 mg; pyridoxine, 10 mg; biotin, 1 mg; choline chloride, 560 mg; ethoxyquin, 125 mg; Mn (from MnSO_4_·H_2_O), 65 mg; Zn (from ZnO), 55 mg; Fe (from FeSO_4_·7H_2_O), 50 mg; Cu (from CuSO_4_·5H_2_O), 8 mg; I (from Ca (IO_3_)2·H_2_O), 1.8 mg; Se, 0.30 mg; Co (from Co_2_O_3_), 0.20 mg; Mo, 0.16 mg.

**Table 2:** Chemical composition (g/kg as is), mineral content (mg/kg, as is), 1-Deoxynojirimycin content (1-DNJ: μg/g), gross energy content (MJ/kg) of full-fat silkworm meal (SWM), and the experimental diets.

	SWM	Experimental Diets			
		C	SWM4	SWM8	SWM12
Dry matter	935	909	911	908	914
Crude protein	378	215	218	210	212
Ether extract	292	67.1	44.0	36.7	48.1
Ash	48.9	97.8	108	106	131
Starch	-	322	302	314	272
Chitin	14.6	0.00	0.20	0.47	1.02
Calcium (Ca)	38.0 ^‡^	25.1	25.1	25.0	25.0
Phosphorous (P)	60.0 ^‡^	3.51	3.51	3.50	3.50
Ca/P	0.63 ^‡^	7.15	7.15	7.15	7.15
1-DNJ	0.76 ^§^	-	0.25	0.431	0.91
Gross energy ^1^	25.2	12.2	12.2	12.2	12.2

C: Control; SWM4, SWM8, and SWM12 are diets corresponding to 4%, 8%, and 12% silkworm chrysalis meal inclusion levels, respectively; ^‡^ Mineral content was reported from Khan et al. [10]; ^§^ Average 1-DNJ content was reported from Vichasilp et al. [7]; ^1^ Analyzed.

**Table 3:** Effect of the dietary inclusion of 0% (Control), 4% (SWM4), 8% (SWM8), and 12% (SWM12) full-fat silkworm (*Bombyx mori*) chrysalis meal in the diet of laying quail on weekly egg production.

	Experimental Diets				RSD ^1^	*p*-Value
	C	SWM4	SWM8	SWM12		
N. of cages ^2^	12	12	12	12		
Egg production (%):						
Week 1	81.4	84.5	88.6	88.3	5.33	0.0579
Week 2	83.5 ^b^	88.3 ^ab^	89.5 ^a^	90.2 ^a^	3.62	0.0093
Week 3	82.6	86.2	89.1	85.0	5.53	0.1955
Week 4	86.9	85.0	86.4	88.9	4.86	0.5314
Week 5	86.5	85.4	86.0	87.7	3.74	0.7089
Week 6	84.8	86.9	85.9	86.0	5.66	0.9205
Week 7	81.5 ᵇ	87.2 ^a^	86.6 ^ab^	85.1 ^ab^	3.72	0.0343
Week 8	85.4	87.8	82.9	86.0	4.00	0.1770
Week 1–8	84.1 ^Bb^	86.3 ^AB^	86.8 ^ABa^	87.0 ^A^	4.71	0.0033

^1^ RSD: Residual standard deviation; ^2^ Five quails/cage; ^a,b^ Means in the same row with different superscript letters differ for *p* < 0.05; ^A,B^ Means in the same row with different superscript letters differ for *p* < 0.01.

**Table 4:** Effect of the dietary inclusion of 0% (Control), 4% (SWM4), 8% (SWM8), and 12% (SWM12) full-fat silkworm (*Bombyx mori*) chrysalis meal in the diet of laying quail on weekly egg weight.

	Experimental Diets				RSD ^1^	*p*-Value
	C	SWM4	SWM8	SWM12		
N. of eggs	2518	2651	2592	2567		
Egg weight (g):						
Week 1	13.1 ^B^	13.2 ^AB^	13.3 ^Aa^	13.1 ^ABb^	1.06	0.0013
Week 2	13.4 ᴬᴮ^ab^	13.5 ᴬᴮᵃ	13.6 ᴬ	13.2 ᴮᵇ	1.05	0.0002
Week 3	13.6 ᴬ	13.6 ᴬ	13.6 ᴬ	13.3 ᴮ	1.02	0.0005
Week 4	13.6 ᴬᵇ	13.6 ᴬᴮᵇᶜ	13.8 ᴬᵃ	13.4 ᴮᶜ	1.02	<0.0001
Week 5	13.6	13.7	13.7	13.6	1.04	0.1751
Week 6	13.7 ᵇᶜ	13.9 ᵃ	13.9 ᵃ	13.6 ᶜ	1.00	0.0027
Week 7	13.7 ᴬᴮ	13.9 ᴬ	13.9 ᴬ	13.5 ᴮ	1.06	<0.0001
Week 8	13.7	13.8	13.9	13.7	0.99	0.1214
Week 1–8	13.6	13.8	13.9	13.5	1.04	0.0587

^1^ RSD: Residual standard deviation; ^a,b,c^ Means in the same row with different superscript letters differ for *p* < 0.05; ^A,B^ Means in the same row with different superscript letters differ for *p* < 0.01.

**Table 5:** Effect of the dietary inclusion of 0% (Control), 4% (SWM4), 8% (SWM8), and 12% (SWM12) full-fat silkworm (*Bombyx mori*) chrysalis meal in the diet of laying quail on weekly feed conversion ratio (feed intake, g/egg produced, g).

	Experimental Diets				RSD ^1^	*p*-Value
	C	SWM4	SWM8	SWM12		
N. of cages ^2^	12	12	12	12		
FCR:						
Week 1	2.58	2.75	2.92	2.77	0.37	0.1812
Week 2	3.52	3.50	3.52	3.62	0.40	0.8656
Week 3	3.19 ^B^	3.33 ^ABb^	3.86 ^Aa^	3.81 ^Aa^	0.40	0.0001
Week 4	3.48 ^b^	3.98 ^ab^	4.01 ^a^	3.98 ^ab^	0.47	0.0201
Week 5	3.05 ^B^	3.46 ^AB^	3.70 ^A^	3.72 ^A^	0.45	0.0016
Week 6	3.22 ^b^	3.66 ^ab^	3.76 ^ab^	3.97 ^a^	0.61	0.0310
Week 7	3.27 ^b^	3.69 ^ab^	3.55 ^ab^	3.95 ^a^	0.57	0.0440
Week 8	3.03 ^Bb^	3.47 ^B^	3.90 ^ABa^	4.67 ^A^	0.73	0.0001
Week 1–8	2.74 ^B^	3.00 ^AB^	3.13 ^A^	3.24 ^A^	0.26	0.0002

^1^ RSD: Residual standard deviation; ^2^ Five quails/cage; ^a,b^ Means in the same row with different superscript letters differ for *p* < 0.05; ^A,B^ Means in the same row with different superscript letters differ for *p* < 0.01.

**Table 6:** Effect of the dietary inclusion of 0% (Control), 4% (SWM4), 8% (SWM8), and 12% (SWM12) full-fat silkworm (*Bombyx mori*) chrysalis meal in the diet of laying quail on weekly egg shape index.

	Experimental Diets				RSD ^1^	*p*-Value
	C	SWM4	SWM8	SWM12		
N. of eggs	2455	2598	2550	2531		
Egg shape index ^2^:						
Week 1	77.9 ^Aa^	77.3 ^ABb^	77.2 ^Bb^	76.7 ^Bc^	3.01	<0.0001
Week 2	77.5 ^A^	76.5 ^B^	76.7 ^B^	76.2 ^B^	2.97	<0.0001
Week 3	77.4 ^A^	76.4 ^Bab^	76.5 ^Ba^	75.9 ^Bb^	2.87	<0.0001
Week 4	77.5 ^A^	76.1 ^B^	76.1 ^B^	76.0 ^B^	2.92	<0.0001
Week 5	76.9 ^A^	76.0 ^Ba^	76.0 ^Ba^	75.3 ^Bb^	2.96	<0.0001
Week 6	76.7 ^Aa^	76.0 ^ABb^	75.7 ^Bbc^	75.3 ^Bc^	3.05	<0.0001
Week 7	76.9 ^A^	75.2 ^BC^	75.6 ^B^	75.0 ^C^	3.01	<0.0001
Week 8	76.6 ^A^	75.8 ^B^	75.6 ^BCa^	74.8 ^Cb^	3.00	<0.0001
Week 1–8	77.2 ^A^	76.3 ^B^	76.3 ^B^	75.8 ^C^	3.01	<0.0001

^1^ RSD: Residual standard deviation; ^2^ Egg shape index: (equatorial diameter/height) × 100; ^a,b,c^ Means in the same row with different superscript letters differ for *p* < 0.05; ^A,B,C^ Means in the same row with different superscript letters differ for *p* < 0.01.

**Table 7:** Effect of the dietary inclusion of 0% (Control), 4% (SWM4), 8% (SWM8), and 12% (SWM12) full-fat silkworm (*Bombyx mori*) chrysalis meal in the diet of laying quail on the physical traits of eggs collected at 63 d of age (week 1).

	Experimental Diets				RSD ^1^	*p*-Value
	C	SWM4	SWM8	SWM12		
N. of eggs	27	16	20	21		
Whole egg:						
Surface area (cm^2^) ^2^	24.5	24.8	24.9	24.4	1.27	0.568
Edible portion (%) ^3^	88.3	88.1	87.9	87.8	1.37	0.595
Shell:						
Weight (g)	1.54	1.58	1.63	1.59	0.18	0.455
Thickness (mm)	0.23	0.21	0.22	0.21	0.02	0.370
Albumen:						
Haugh unit ^4^	96.7	100	94.8	96.9	9.56	0.403
Yolk:						
Color FAN ^5^	14.4 ᵃ	13.8 ᵇ	14.3 ᵃᵇ	13.9 ᵇ	0.65	0.008

^1^ RSD: Residual standard deviation; ^2^ Surface area: [(Egg weight)^0.7056^] × 3.9782; ^3^ Edible portion: (Albumen weight + Yolk weight)/Egg weight × 100; ^4^ Haugh unit [21]; ^5^ Roche yolk color fan [22]; ^a,b^ Means in the same row with different superscript letters differ for *p* < 0.05.

**Table 8:** Effect of the dietary inclusion of 0% (Control), 4% (SWM4), 8% (SWM8), and 12% (SWM12) full-fat silkworm (*Bombyx mori*) chrysalis meal in the diet of laying quail on the physical traits of eggs collected at 119 d of age (week 8).

	Experimental Diets				RSD ^1^	*p*-Value
	C	SWM4	SWM8	SWM12		
N. of eggs	84	84	84	84		
Whole egg:						
Surface area (cm^2^) ^2^	25.1	25.4	25.5	25.0	1.35	0.0564
Edible portion (%) ^3^	87.6 ᴮ	88.3 ᴬᴮ	88.4 ᴬ	88.7 ᴬ	1.58	<0.0001
Yolk/Albumen	0.51	0.53	0.51	0.51	0.06	0.1818
Shell:						
Weight (g)	1.68 ᴬ	1.62 ᴬᴮᵃ	1.61 ᴬᴮ	1.52 ᴮᵇ	0.22	0.0001
Thickness (mm)	0.26	0.26	0.25	0.26	0.04	0.1652
Percentage (%) ^4^	12.4 ᴬ	11.7 ᴬᴮ	11.6 ᴮ	11.3 ᴮ	1.58	<0.0001
Albumen:						
Weight (g)	7.91	8.02	8.15	7.98	0.71	0.1502
Percentage % ^5^	58.1	57.9	58.6	59.0	2.73	0.0471
pH	9.07 ᵇ	9.10 ᵃᵇ	9.07 ᵇ	9.13 ᵃ	0.15	0.0125
Haugh Unit ^6^	94.7	94.9	94.4	94.0	2.68	0.2033
Yolk:						
Weight (g)	4.03	4.20	4.15	4.04	0.48	0.0502
Percentage (%) ^7^	29.6	30.3	29.8	29.8	2.40	0.1846
Color FAN ^8^	5.01 ᴬ	4.83 ᴬᴮ	4.62 ᴮ	4.99 ᴬ	0.60	<0.0001

^1^ RSD: Residual standard deviation; ^2^ Surface area: [(Egg weight)^0.7056^] × 3.9782; ^3^ Edible portion percentage: (Albumen weight + Yolk weight)/Egg weight × 100; ^4^ Shell percentage: (Shell weight/Egg weight) × 100; ^5^ Albumen percentage: (Albumen weight/Egg weight) × 100; ^6^ Haugh unit [21]; ^7^ Yolk percentage: (Yolk weight/Egg weight) × 100; ^8^ Roche yolk color fan [22]; ^a,b^ Means in the same row with different superscript letters differ for *p* < 0.05; ^A,B^ Means in the same row with different superscript letters differ for *p* < 0.01.

## Data Availability

The data sets analyzed in the present study are available from the corresponding author upon request.

## References

[1] FAO, IFAD, UNICEF, WFP, WHO The State of Food Security and Nutrition in the World 2022. https://www.fao.org/3/cc0639en/online/cc0639en.html.

[2] OECD-FAO Agricultural Outlook 2021–2030. https://stats.oecd.org/viewhtml.aspx?QueryId=107144&vh=0000&vf=0&l&il=&lang=en.

[3] International Feed Industry Federation (IFIF). https://ifif.org/.

[4] National Research Council (NRC) (1994). Subcommittee on Poultry Nutrition 1994. Nutrient requirements of ring-necked pheasants, Japanese quail, and bobwhite quail. Nutrient Requirements of Poultry.

[5] Hăbeanu M., Gheorghe A., Mihalcea T. (2023). Nutritional Value of Silkworm Pupae (*Bombyx mori*) with Emphases on Fatty Acids Profile and Their Potential Applications for Humans and Animals. Insects.

[6] Dalle Zotte A., Singh Y., Squartini A., Stevanato P., Cappellozza S., Kovitvadhi A., Subaneg S., Bertelli D., Cullere M. (2021). Effect of a dietary inclusion of full-fat or defatted silkworm pupa meal on the nutrient digestibility and faecal microbiome of fattening quails. Animal.

[7] Vichasilp C., Wechavitan P., Poungchompu O., Wiwacharn P. (2016). Extraction of 1-Deoxynojirimycin (DNJ) from silkworms as functional foods for lower postprandial glucose. Int. Proc. Chem. Biol. Environ. Eng..

[8] Zsedely E., Cullere M., Takacs G., Herman Z., Szalai K., Singh Y., Dalle Zotte A. (2023). Dietary Inclusion of Defatted Silkworm (*Bombyx mori* L.) Pupa Meal for Broiler Chickens at Different Ages: Growth Performance, Carcass and Meat Quality Traits. Animals.

[9] Miah M.Y., Singh Y., Cullere M., Tenti S., Dalle Zotte A. (2020). Effect of dietary supplementation with full-fat silkworm (*Bombyx mori* L.) chrysalis meal on growth performance and meat quality of Rhode Island Red× Fayoumi crossbred chickens. Ital. J. Anim. Sci..

[10] Khan S., Khan R.U., Alam W., Sultan A. (2018). Evaluating the nutritive profile of three insect meals and their effects to replace soya bean in broiler diet. J. Anim. Physiol. Anim. Nutr..

[11] Rafiullah, Khan S., Khan R.U., Ullah Q. (2020). Does the gradual replacement of spent silkworm (*Bombyx mori*) pupae affect the performance, blood metabolites and gut functions in White Leghorn laying hens?. Res. Vet. Sci..

[12] Ullah R., Khan S., Khan N.A., Mobashar M., Sultan A., Ahmad N., Lohakare J. (2017). Replacement of soybean meal with silkworm meal in the diets of white leghorn layers and effects on performance, apparent total tract digestibility, blood profile and egg quality. Int. J. Vet. Health Sci. Res..

[13] Rahmasari R., Sumiati S., Astuti D.A. (2014). The effect of silkworm pupae (*Bombyx mori*) meal to substitute fish meal on production and physical quality of quail eggs (*Cortunix cortunix japonica*). J. Indones. Trop. Anim. Agric..

[14] Dalle Zotte A., Singh Y., Michiels J., Cullere M. (2019). Black soldier fly (*Hermetia illucens*) as dietary source for laying quails: Live performance, and egg physico-chemical quality, sensory profile and storage stability. Animals.

[15] AOAC (2000). Official Methods of Analysis.

[16] Zhang J., Zhu K.Y. (2006). Characterization of a chitin synthase cDNA and its increased mRNA level associated with decreased chitin synthesis in Anopheles quadrimaculatus exposed to diflubenzuron. Insect Biochem. Mol. Biol..

[17] Woods M.J., Cullere M., Van Emmenes L., Vincenzi S., Pieterse E., Hoffman L.C., Zotte A.D. (2019). *Hermetia illucens* larvae reared on different substrates in broiler quail diets: Effect on apparent digestibility, feed-choice and growth performance. J. Insects Food Feed.

[18] EC Commission Directive 98/64/EC of 3 September 1998 Establishing Community Methods of Analysis for the Determination of Amino Acids, Crude Oils and Fats, and Olaquindox in Feeding Stuffs and Amending Directive 71/393/EEC. https://publications.europa.eu/en/publication-detail/-/publication/856db9b7-6f1d-4d6e-a778-c766c9fa1776.

[19] Wang L., Peng J., Wang X., Zhu X., Cheng B., Gao J., Jiang M., Bai G., Hou Y. (2012). Carboxymethylcellulose sodium improves the pharmacodynamics of 1-deoxynojirimycin by changing its absorption characteristics and pharmacokinetics in rats. Die Pharm.-Int. J. Pharm. Sci..

[20] ISO International Organization for Standardization. Animal Feeding Stuffs, Animal Products and Faeces or Urine. Determination of Gross Calorific Value-Bomb Calorimetric Method; Reference Number 9831, Prepared by Technical Committee ISO/TC 34, Agricultural Food Products, Subcommittee SC 10, Animal Feeding Stuffs. https://www.iso.org/obp/ui/#iso:std:iso:9831:ed-1:v1:en..

[21] Haugh R.R. (1973). A new method for determining the quality of an egg. U.S. Poult. Mag..

[22] Vuilleumier J.P. (1969). The ‘Roche Yolk Colour Fan—An Instrument for Measuring Yolk Colour. Poult. Sci..

[23] Khatun R., Azmal S.A., Sarker M.S.K., Rashid M.A., Hussain M.A., Miah M.Y. (2005). Effect of silkworm pupae on the growth and egg production performance of Rhode Island Red (RIR) pure line. Int. J. Poult. Sci..

[24] Wathes D.C., Abayasekara D.R.E., Aitken R.J. (2007). Polyunsaturated fatty acids in male and female reproduction. Biol. Reprod..

[25] Abayasekara D.R.E., Wathes D.C. (1999). Effects of altering dietary fatty acid composition on prostaglandin synthesis and fertility. Prostaglandins Leukot. Essent. Fat. Acids.

[26] Hrnčár C., Hanusova E., Hanus A., Bujko J. (2014). Effect of genotype on egg quality characteristics of Japanese quail (*Coturnix japonica*). Slovak J. Anim. Sci..

[27] Cullere M., Tasoniero G., Giaccone V., Miotti-Scapin R., Claeys E., De Smet S., Dalle Zotte A. (2016). Black soldier fly as dietary protein source for broiler quails: Apparent digestibility, excreta microbial load, feed choice, performance, carcass and meat traits. Animal.

[28] Hatab M.H., Ibrahim N.S., Sayed W.A., Sabic E.M. (2020). Potential value of using insect meal as an alternative protein source for Japanese quail diet. Braz. J. Poult. Sci..

[29] Duman M., Sekeroglu A., Yildirim A., Eleroglu H., Camci O. (2016). Relation between egg shape index and egg quality characteristics. Eur. Poult. Sci..

[30] Minvielle F., Oguz Y. (2002). Effects of genetics and breeding on egg quality of Japanese quail. World’s Poult. Sci. J..

[31] Dudusola I., Bashiru H.A., Adeleke O.E. (2020). Effect of laying age and plumage colour on internal and external quality characteristics of noiler chicken eggs. Slovak J. Anim. Sci..

[32] Cullere M., Singh Y., Pellattiero E., Berzuini S., Galasso I., Clemente C., Dalle Zotte A. (2023). Effect of dietary inclusion of *Camelina sativa* cake into quail diet on live performance, carcass traits and meat quality. Poult. Sci..

[33] Gernat A.G. (2001). The effect of using different levels of shrimp meal in laying hen diets. Poult. Sci..

[34] Secci G., Bovera F., Parisi G., Moniello G. (2020). Quality of eggs and albumen technological properties as affected by *Hermetia illucens* larvae meal in hens’ diet and hen age. Animals.

[35] Caner C., Yüceer M. (2015). Efficacy of various protein-based coating on enhancing the shelf life of fresh eggs during storage. Poult. Sci..

[36] Chieco C., Morrone L., Bertazza G., Cappellozza S., Saviane A., Gai F., Di Virgilio N., Rossi F. (2019). The effect of strain and rearing medium on the chemical composition, fatty acid profile and carotenoid content in silkworm (*Bombyx mori*) pupae. Animals.

